# Intracerebral Hemorrhages in Adults with Community Associated Bacterial Meningitis in Adults: Should We Reconsider Anticoagulant Therapy?

**DOI:** 10.1371/journal.pone.0045271

**Published:** 2012-09-13

**Authors:** Barry B. Mook-Kanamori, Daan Fritz, Matthijs C. Brouwer, Arie van der Ende, Diederik van de Beek

**Affiliations:** 1 Department of Neurology, Center of Infection and Immunity Amsterdam, Academic Medical Center, University of Amsterdam, Amsterdam, The Netherlands; 2 Department of Medical Microbiology, Center of Infection and Immunity Amsterdam, Academic Medical Center, University of Amsterdam, Amsterdam, The Netherlands; 3 The Netherlands Reference Laboratory for Bacterial Meningitis, Center of Infection and Immunity Amsterdam, Academic Medical Center, University of Amsterdam, Amsterdam, The Netherlands; University of Iowa Carver College of Medicine, United States of America

## Abstract

**Objective:**

To study the incidence, clinical presentation and outcome of intracranial hemorrhagic complications in adult patients with community associated bacterial meningitis.

**Methods:**

Nationwide prospective cohort study from all hospitals in the Netherlands, from 1 March 2006, through 31 December 2010.

**Results:**

Of the 860 episodes of bacterial meningitis that were included, 24 were diagnosed with intracranial hemorrhagic complications: 8 upon presentation and 16 during clinical course. Clinical presentation between patients with or without intracranial hemorrhage was similar. Causative bacteria were *Streptococcus pneumoniae* in 16 patients (67%), *Staphylococcus aureus* in 5 (21%), *Pseudomonas aeruginosa* and *Listeria monocytogenes* both in 1 patient (4%). Occurrence of intracranial hemorrhage was associated with death (63% vs. 15%, P<0.001) and unfavorable outcome (94% vs. 34%, P<0.001). The use of anticoagulants on admission was associated with a higher incidence of intracranial hemorrhages (odds ratio 5.84, 95% confidence interval 2.17–15.76).

**Conclusion:**

Intracranial hemorrhage is a rare but devastating complication in patients with community-associated bacterial meningitis. Since anticoagulant therapy use is associated with increased risk for intracranial hemorrhage, physicians may consider reversing or temporarily discontinuing anticoagulation in patients with bacterial meningitis.

## Introduction

Bacterial meningitis is a life threatening disease with an incidence of 2 cases per 100,000 adults [1], [2]. *Streptococcus pneumoniae* and *Neisseria meningitidis* together cause 80% of all cases, leading to a mortality of up to 37% and 13%, respectively [1], [3], [4]. Of those patients who survive, up to 50% have neurological sequelae, including hearing loss and neuropsychological deficits [1], [4], [5]. One of the major causes of mortality and neurological sequelae is the development of cerebrovascular complications, of which cerebral ischemia is most frequently reported [1], [6], [7], [8]. Intracranial hemorrhages have been described as an uncommon complication of meningitis occurring in 2–9% of cases [6], [7], [9]. In this study, we investigated the prevalence, characteristics and outcome of patients who develop hemorrhages as a complication of community-associated bacterial meningitis.

## Methods

In this prospective nationwide cohort study, patients older than 16 years were included who were listed in the database of the Netherlands Reference Laboratory for Bacterial Meningitis (NRLBM) in the period from March 2006 through December 2010. Ninety percent of all patients with cerebrospinal fluid (CSF) culture-positive bacterial meningitis in the Netherlands are registered by the NRLBM, which supplied daily updates of the names of the hospitals where patients had been admitted with bacterial meningitis during the previous 2–6 days. The treating physician was contacted and informed consent was obtained from all participating patients or their legally authorized representatives. Patients with bacterial meningitis who were not registered with the NRLBM could also be included if physicians contacted us directly. Patients with negative CSF cultures were only included if the clinical presentation was consistent with bacterial meningitis and the CSF analysis demonstrated at least 1 individual predictor of bacterial meningitis (defined as a glucose level of less than 34 mg/dL [1.9 mmol/L], a ratio of CSF glucose to blood glucose of less than 0.23, a protein level of more than 220 mg/dL, or a leukocyte count of more than 2,000/mm^3^) [10]. Patients with negative CSF cultures but positive CSF gram stains were also included. All patients with a hospital associated meningitis, recent neuro-trauma or neurosurgical procedure were excluded from analysis. These cases of meningitis have different pathophysiological mechanisms than cases associated with the community setting, and are caused by a different spectrum of microorganisms [11].

Upon discharge, patients underwent a neurological examination and outcome was assessed using the Glasgow Outcome Scale, a well validated measurement scale with scores ranging from 1 (death) to 5 (good recovery) [12]. A score of 1 on this scale indicates death; a score of 2, a vegetative state (the patient is unable to interact with the environment); a score of 3, severe disability (the patient is unable to live independently but can follow commands); a score of 4, moderate disability (the patient is capable of living independently but unable to return to work or school); and a score of 5, mild or no disability (the patient is able to return to work or school). A favorable outcome was defined as a score of 5, and an unfavorable outcome was defined as a score of 1 to 4. The study was approved by the ethical review committee of participating hospitals.

Patients' data was collected by means of a digital Clinical Record From (CRF) by the treating physician. Additional information, including the use of anticoagulant or platelet aggregation therapy, was gathered from discharge letters. Patients were classified as having an intracranial hemorrhagic complication if reported by the treating physician and cranial imaging confirmed the presence of intracranial blood. Neuroimaging was obtained from all patients with an intracranial hemorrhage and was reassessed by two investigators (B.M-K., D.F.) to determine the presence, type and distribution of the hemorrhage. To check for underreporting by physicians, we evaluated 150 consecutive patients included in the cohort who underwent cranial imaging and were not reported to have intracranial hemorrhages by the physician; none of these patients had cerebral hemorrhages.

Population description was performed using medians and interquartile ranges. Differences between episodes of bacterial meningitis with and without hemorrhages were assessed using a Mann-Whitney *U*-test regarding continuous variables, and a χ^2^-test or Fisher's exact test regarding dichotomous variables. We used logistic regression analysis to calculate odds ratio (OR) and 95% confidence interval (CI) to assess the strength of any observed associations. All statistical tests were 2-tailed, and a *p* value of <0.05 was considered to be significant. All analyses were executed using SPSS software, version 16.0.

## Results

**Figure 1:**
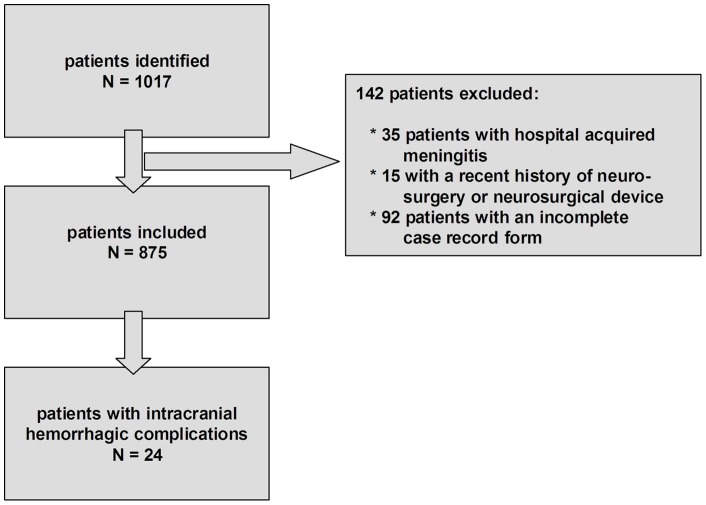
Selection of patients.

**Figure 2:**
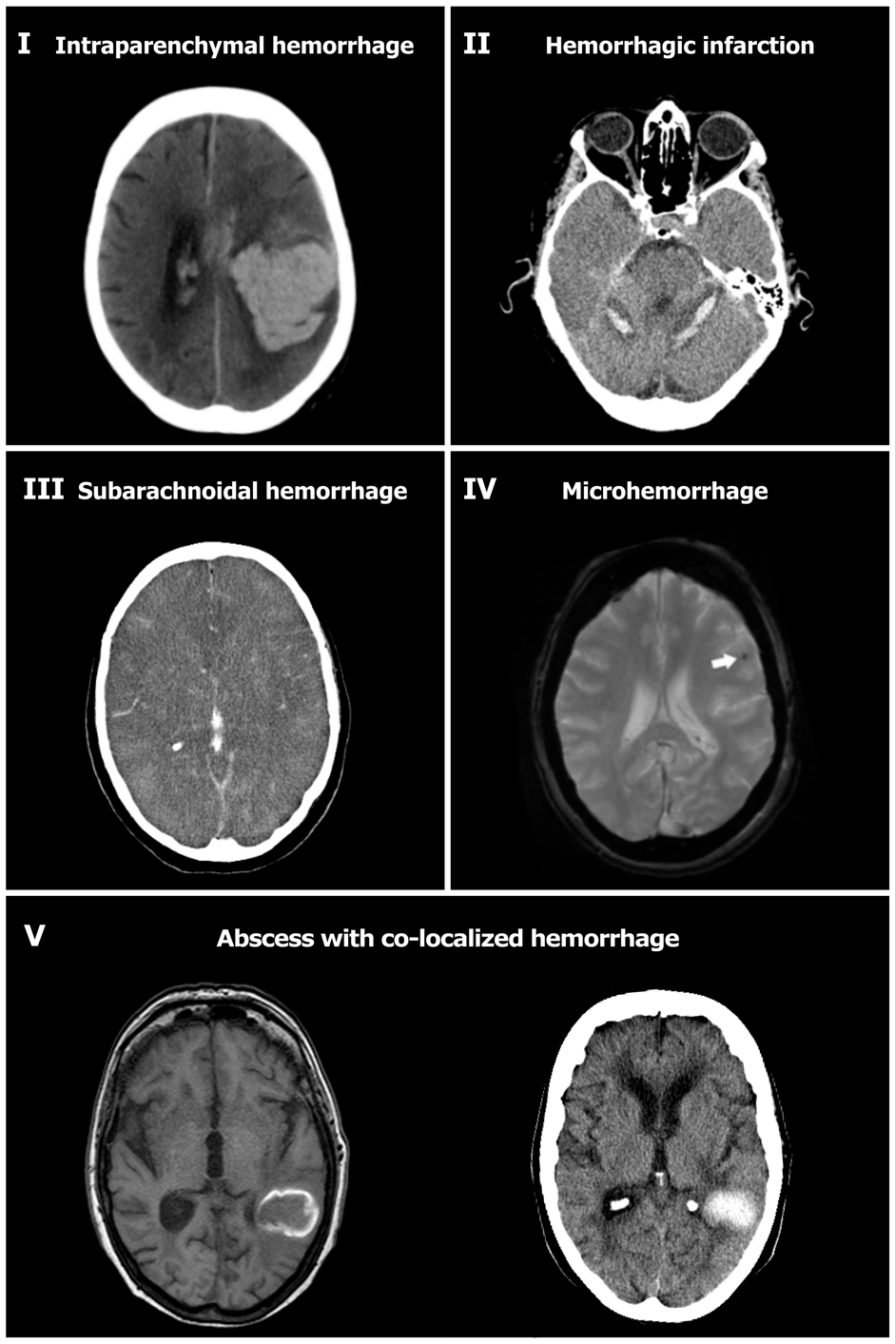
Types of intracranial hemorrhagic complications encountered in bacterial meningitis patients. Intraparenchymal hemorrhage in left parietal lobe (I); hemorrhagic infarction (II); subarachnoidal hemorrhage (III); micro-hemorrhages (IV, arrow depicts location of hemorrhage; MRI-gradient echo); Abscess formation and subsequent hemorrhagic transformation; panel V depicts MRI T-1 with gadolinium, and a CT-scan 5 days later).

One-thousand-and-two patients with bacterial meningitis were identified by the national reference laboratory (Figure 1). Of the initial 1002, 142 were excluded –35 with hospital associated bacterial meningitis infection; 15 with recent neurosurgical procedure or neurosurgical device; 92 with an incomplete case record form. Thus, a total of 860 patients were included in the final analysis. The causative species were *S. pneumoniae* in 576 episodes (67%), *N. meningitidis* in 96 episodes (11%), and other bacteria in 188 episodes (22%). Cerebral hemorrhage was diagnosed in 24 of 860 (2.8%) patients (Table 1). Eight patients were diagnosed with cerebral hemorrhage on presentation and 16 during clinical course (median time to detection of hemorrhage 8 days; range, 1–48 days). Hemorrhages were classified as parenchymal in 10 patients, subarachnoid in 5, microhemorrhages in 3, secondary hemorrhagic transformation following cerebral infarction in 4, and hemorrhages co-localizing with a cerebral abscess in 2 (Figure 2). Predisposing conditions for bacterial meningitis were present in 16 of the 24 (67%) patients, of which the most common were an immunocompromised state in 42% patients, otitis/sinusitis and infective endocarditis each in 17%, and pneumonia in 13%.

Of the eight patients diagnosed with intracranial hemorrhagic upon admission, focal neurological deficits were present in only one patient. Clinical presentation did not differentiate between patients with or without intracranial hemorrhage, although patients presenting with intracranial hemorrhage were more likely to have an extensor plantar reflex (3 of 7 [43%] vs. 110 of 735 [15%]; P = 0.041). During admission an additional 16 patients were diagnosed with cerebral hemorrhage. In these patients the cerebral hemorrhage was identified on cranial imaging after the patients developed a progressive impairment of consciousness (in 13 patients), developed focal neurological deficit (in 3 patients), showed no clinical improvement (in 2 patients), or developed a status epilepticus (in 1 patient).

**Table 1:** Clinical and laboratory characteristics in bacterial meningitis episodes complicated by intracranial hemorrhage^a^.

Clinical characteristics	n/N (%)	Clinical characteristics	n/N (%)
Median age, y	64 (50–74)	Focal neurologic deficits	11/20 (55)
Male	13/24 (54)	Cranial nerve palsy	4/23 (17)
Predisposing factors for meningitis	16/24 (67)	Aphasia	3/13 (23)
Otitis or sinusitis	4/24 (17)	Hemiparesis	4/20 (20)
Pneumonia	3/24 (13)	Blood values	
Endocarditis	4/24 (17)	Trombopenia (<150x10^9^/L)	2/7 (29)
Immunocompromised state^b^	10/24 (42)	CSF values	
Medication on admission		White blood cells (cells/mm^3)^	889 (267–2716)
Anticoagulant therapy^c^	6/24 (25)	<1000/mm^3^	16 (67)
Platelet aggregation inhibitors^d^	5/24 (21)	Protein, g/L	2.99 (1.20–5.62)
Symptoms and signs on admission		CSF: blood glucose ratio	0.03 (0.00–0.43)
Symptoms <24 h	8/20 (40)	Microbiological findings	
Headache	11/19 (58)	Positive Gram stain^e^	16/23 (70)
Neck stiffness	17/23 (74)	Positive blood culture	18/22 (82)
Seizures	1/22 (5)	CSF culture	
Temperature ≥38^o^C	17 (71)	*Streptococcus pneumoniae*	16 (67)
Triad (neck-stiffness, fever,altered mental status)	13 (54)	*Staphylococcus aureus*	5 (21)
Score on Glasgow Coma Scale (GCS)	12 (10–13)	*Pseudomonas aeruginosa*	1 (4)
Altered mental status (GCS <14)	20 (83)	*Listeria monocytogenes*	1 (4)
Coma (GCS <8)	1 (4)	Negative	1 (4)

a Data are number/number evaluated (%) or median (interquartile range).

b Immunocompromise was defined by the use of immunosuppressive drugs (1), a history of splenectomy (1), or the presence of diabetes mellitus (4) or alcoholism (4), as well as patients infected with the human immunodeficiency virus (HIV)(1).

c Five patients were using oral anticoagulants (coumarin derivatives); 2 were using subcutaneous low molecular weight heparin (nadroparin) in therapeutic doses.

d Four patients were using acetylsalicylic acid; 1 was using clopidogrel.

e Gram-positive cocci in 14 (58%), Gram-positive rods in 0, Gram-negative cocci in 1 (4%), and Gram-negative rods in 1 (4%).

Of the 639 patients of which information regarding the use of anticoagulant therapy could be obtained, 41 patients (6%) were using anticoagulant therapy upon admission. Of these patients, 6 patients (15%) developed intracranial hemorrhage (Table 2). Indication for anticoagulation therapy in these 6 patients were history of venous thrombotic events in 3 patients, antithrombin-III deficiency and factor V leiden-mutations in 1 patient each, prosthetic heart-valves in 2 patients, atrial fibrillation in 3 patients. At the time of developing the intracranial hemorrhage all six patients had prolonged coagulation times and/or documented use of anticoagulant therapy. Two patients were diagnosed with hemorrhages in the first week of admission, 1 patient in the second week and 3 patients after more than 2 weeks after admission. Anticoagulant therapy was converted and stopped in all patients after intracranial hemorrhage was diagnosed. The risk of an intracranial hemorrhagic complication in patients using anticoagulants was significantly higher compared to patients who did not use anticoagulants (odds ratio 5.84, 95% confidence interval 2.17–15.76). The risk of intracranial hemorrhagic complications was not increased in patients using platelet aggregation therapy or patients with a thrombocytopenia (odds ratio 2.00, 95% confidence interval 0.72–5.54).

**Table 2:** Clinical characteristics of patients with bacterial meningitis with intracranial hemorrhagic complications vs. patients without hemorrhagic complications^a^.

Clinical characteristics	Patients with brain hemorrhage (n = 24)	Patients without brain hemorrhage (n = 836)	p-value
Median age, y	64 (50–74)	60 (44–69)	0.207
Predisposing conditions	16/24 (67)	494/831 (59)	0.477
Endocarditis	4/24 (17)	8/808 (1)	<0.001^c^
Medication upon admission			
Anticoagulant therapy^b^	6/23 (25)	35/615 (6)	0.002^c^
Platelet aggregation inhibitors	5/24 (21)	75/615 (12)	0.193^c^
Symptoms and signs on admission			
Seizures	1/22 (5)	48/810 (6)	1.00^c^
Score on Glasgow Coma Scale	12 (10–13)	11 (9–14)	0.482
Altered mental status (GCS <14)	20/24 (83)	602/836 (72)	0.222
Coma (GCS <8)	1/24 (4)	111/836 (13)	0.350^c^
Focal neurologic deficits	9/24 (38)	224/836 (27)	0.245
Cranial nerve palsy	4/24 (17)	63/769 (8)	0.122^c^

a Data are number/number evaluated (%) or median (interquartile range).

b Five patients were using oral anticoagulants (coumarin derivatives); 2 were using subcutaneous low molecular weight heparin (nadroparin) in therapeutic doses.

c Fisher exact test.

A lumbar puncture was performed in all 24 patients. The CSF of all patients had either a positive culture and/or at least one individual CSF finding predictive of bacterial meningitis (glucose level less that 34 mg/dL [1.9 mmol/L], a ratio of CSF glucose to blood glucose of less than 0.23, a protein level of more than 220 mg/dL, or a leukocyte count of more than 2,000/mm^3^) [10]. The CSF leukocyte count was lower in patients with intracranial hemorrhage *upon* or *during* admission than in patients without intracranial hemorrhagic complications (67% of patients under 1000 leukocytes/mm^3^ vs. 30%, P<0.001). Other CSF parameters did not significantly differ. CSF cultures revealed *S. pneumoniae* in 16 of 24 patients (67%), *Staphylococcus aureus* in 5 (21%), *Pseudomonas aeruginosa* and *Listeria monocytogenes* both in 1 patient (4%). One patient had a negative CSF culture but had a positive Gram stain. Four patients fulfilled the Dukes criteria for an infective endocarditis (17%) – in all cases the causative agents was *S. aureus*.

Neurosurgical interventions were performed in 2 patients – one HIV–positive patient with *S. aureus* meningitis, which was complicated by a subarachnoidal hemorrhage, underwent a bilateral frontotemporal craniotomy and died 10 days after the procedure following rebleeding from a mycotic aneurysm. Another patient with a *S. pneumoniae* meningitis and microhemorrhage in the left hemisphere developed a hydrocephalus and deteriorating consciousness. An external ventricular drain was placed and she partially recovered with persisting cranial nerve palsies and ataxia.

Occurrence of intracranial hemorrhage was associated with a higher rate of mortality (63% vs. 15s P<0.001) and unfavorable outcome (96% vs. 34%, P<0.001) than in patients without intracranial hemorrhagic complication (Table 3). There were significantly more neurological sequelae among the survivors (78% vs. 14%, P<0.001).

**Table 3:** Complications and outcome in adults with vs. without intracranial hemorrhagic complicating bacterial meningitis^a^.

Characteristic	Episodes with Brain Hemorrhage (n = 24)	Episodes without Brain Hemorrhage (n = 836)	p-value
Complications			
Impairment of consciousness	23/24 (96)	447/825 (54)	<0,001
Focal neurologic deficits	11/20 (56)	163/796 (21)	<0.001
Systemic complications^b^	20/24 (83)	302/824 (37)	<0.001
Glasgow Outcome Scale			
1. Death	15/24 (63)	129/836 (15)	<0.001^d^
2. Vegetative state	0/24	1/836 (0.1)	0.99^d^
3. Severely disabled	2/24 (8)	35/836 (4)	0.561^d^
4. Moderately disabled	6/24 (25)	116/836 (14)	0.135^d^
5. Good recovery	1/24 (4)	555/836 (66)	<0.001^d^
Neurologic sequelae	7/9 (78)^c^	99/707 (14)	<0.001^d^

a Data are number/number evaluated (%).

b Systemic complications included respiratory failure and circulatory shock (58% and 67%, respectively).

c Neurologic sequelae in patients with intracranial hemorrhage include hemiparesis (4), cognitive impairment (3), cranial nerve palsy (3) and ataxia (3).

d Fisher exact test.

## Discussion

Our study shows that intracranial hemorrhages are a severe complication of bacterial meningitis, occurring in about 3% of adults. Two previous case series from tertiary referral centers have reported rates varying from 2% to 9% [7], [13]. Our study was performed nationwide and, therefore, we are able to study a representative sample of adults with acute bacterial meningitis. Patients with intracranial hemorrhages were at high risk for unfavorable outcome (96%) and death (67%).

A high proportion of the patients who developed intracranial hemorrhagic complications were using anticoagulant therapy when they developed meningitis (25%), of which most were diagnosed with intracranial hemorrhages during admission. Conversely, our data shows a 5-fold increased risk of developing intracranial hemorrhage if the patient with bacterial meningitis uses anticoagulant therapy. Therefore, the question is whether anticoagulant therapy should be discontinued once a patient has been diagnosed with bacterial meningitis. Or, practically speaking, after a lumbar puncture is performed upon admission (for which prior reversal of anticoagulant therapy is necessary), is it advisable to immediately resume anticoagulant therapy? Previous studies on the effect of anticoagulant therapies in bacterial meningitis showed a higher rate of hemorrhagic complications and mortality [14], [15]. In a retrospective analysis of several open-label, placebo controlled and compassionate-use trials of activated protein C (APC), intracranial hemorrhage was seen in 6% of patients treated with APC compared to 3% of patients receiving placebo or no APC. Adjunctive treatment with heparin was examined in a clinical trial in which 15 patients were randomized to a heparin or control treatments; 4 of 7 patients (57%) receiving heparin died compared to 2 of 8 patients (25%) receiving control treatment. No studies have investigated the risk or benefit of discontinuing anticoagulation therapy once bacterial meningitis has been diagnosed. In the light of our present findings, we suggest that physicians weigh the risks of intracranial hemorrhage against the risks of the pro-thrombotic state for which the anticoagulant was prescribed, and consider discontinuation of therapeutic anticoagulant treatment until the patient has recovered from the acute phase of the bacterial meningitis episode (*e.g*., 2–3 weeks).

A relative high proportion of patients with cerebral hemorrhage had *S. aureus* meningitis. *S. aureus* is a rare cause of community-associated bacterial meningitis and has previously been associated with endocarditis and cerebral abscesses [16], [17]. In our cohort of 860 patients 13 patients (1.5%) had *S. aureus* meningitis, five of whom developed intracranial hemorrhages (38%). Of these five patients with *S. aureus* meningitis and intracranial hemorrhagic complications, four fulfilled the Dukes criteria for having infective endocarditis, two had cerebral abscesses, and two were using anticoagulant therapy. The occurrence of ischemic stroke in patients in *S. aureus* infective endocarditis has previously been shown to be between 22% and 34%.[18], [19] Intracranial hemorrhages in *S. aureus* infective endocarditis are thought to be caused by hemorrhagic transformations of cerebral ischemia and occur in 3–17% of patients [18], [19]. Debate is still ongoing regarding the benefits and potential risks of anticoagulant therapy in these patients [20], [21], [22]. The higher rate of intracranial hemorrhages in patients with *S. aureus* meningitis and infective endocarditis (*vs.* those patients with infective endocarditis alone), suggests that anticoagulant therapy should not be prescribed in these patients. The increased risk for intracranial hemorrhage in patients with bacterial meningitis using anticoagulant therapy remains marked (odds ratio 4.62, 95% confidence interval 1.45–14.71) even when patients with endocarditis are excluded from the analysis. Two patients with *S. aureus* meningitis had secondary haemorrhages that co-localized to the site of the abscess.

The rate of mortality and unfavorable outcome in patients with bacterial meningitis and intracranial hemorrhage is high (65% and 95% respectively). However, it is improbable that all observed intracranial hemorrhages were symptomatic and contributed equally to the clinical outcome, which is likely to be also determined by the severity of the meningitis itself, as well as the occurrence of other systemic or neurologic complications. Three of the patients with intracranial hemorrhage had a intracranial abscess, four patients had cerebral infarctions and two had a hydrocephalus, which are all associated with higher mortality and unfavorable outcome [8], [16], [23]. The underlying pathophysiology of cerebrovascular complications during bacterial meningitis (infarct, venous thrombosis and hemorrhages) is largely unknown but may share common pathophysiological mechanisms: first, there is a dysregulation of both the coagulation and fibrinolytic pathways, not only systemically but also locally in the central nervous system compartment [24], [25], [26]. Second, vascular endothelial cell swelling and activation is a common finding in bacterial meningitis, which leads to release of procoagulant factors and pro-inflammatory cytokines, causing further endothelial activation and swelling [27]. Finally, vasculitis has been proposed as a possible mechanism of cerebral infarction, largely supported by autopsy studies from the 1930′s through 1960′s and angiographic description of segmental arterial narrowing in patients with pneumococcal meningitis, although a recent series of autopsies suggested that vascular complications may also occur in non-vasculitic areas [28]. Massive clotting hypothetically may result in the local depletion of coagulation factors, which together with microvascular damage, vasculitis and cerebral infarction, might lead to the observed manifest cerebral hemorrhages [28].

Our study has several limitations. First, not all patients in the cohort underwent cranial neuroimaging (14% did not undergo imaging). Thus, intracranial hemorrhagic complications may have been missed. Second, only patients who underwent a lumbar puncture and fulfilled CSF criteria for bacterial meningitis were included in the cohort. Patients presenting with a space occupying lesion such as a large intracerebral hemorrhage on initial neuroimaging are not likely to have undergone a lumbar puncture and will therefore not have been included in this cohort. Likewise, those patients whose anticoagulation therapy was not reversed also are unlikely to have undergone a lumbar puncture and are not included in the cohort. These factors may have led to an underestimation of the incidence of intracranial hemorrhage and the use of anticoagulant therapy in patients with bacterial meningitis.

We conclude that intracranial hemorrhage is a rare but devastating complication in patients with community-associated bacterial meningitis. Since anticoagulant therapy use is associated with increased risk for intracranial hemorrhage, physicians may consider reversing or temporarily discontinuing anticoagulation in these patients.
